# Intestinal FDG-PET/CT imaging of an Eritrean with schistosomiasis seen in Denmark

**DOI:** 10.1186/s41824-019-0064-4

**Published:** 2019-10-07

**Authors:** Ata Daghigh, Julie Marie Grüner, Peter Mørup

**Affiliations:** Department of Clinical Physiology and Nuclear Medicine, Zealand University Hospital, Lykkebækvej 1, 4600 Køge, Denmark

**Keywords:** Intestinal parasite, *Schistosoma mansoni*, FDG-PET/CT

## Abstract

**Background:**

Schistosomiasis is one of the most common parasitic diseases in subtropical and tropical areas and still is considered of public health significance. This disease affects about 200 million people around the world. Intestinal schistosomiasis is mainly diagnosed by parasitological, serological, and molecular methods.

**Case presentation:**

A 36-year-old Eritrean man who had lived in Denmark for the past 3 years presented to the hospital with 4 months’ history of abdominal pain, back pain, and weight loss of 12 kg. He underwent 18F-FDG-PET/CT scanning. The scan findings were consistent with schistosomiasis, which were confirmed by serological and pathological tests.

**Conclusion:**

PET/CT is a common modality neither to detect schistosomes nor to diagnose schistosomiasis. A presumptive diagnosis can be made based on coincidence of high FDG uptake in visceral lymph nodes below the diaphragm and in relation to abdominal viscera, travel history suggestive of schistosome infection, and exclusion of other causes of abdominal pain.

## Background

Five schistosome species can cause infection in humans (Table 1). The incubation period for patients with acute schistosomiasis is usually 14–84 days; however, many people are asymptomatic and have subclinical disease during both acute and chronic stages of infection. Persons with acute infection (also known as Katayama syndrome) may present with rash, fever, headache, myalgia, and respiratory symptoms. Often eosinophilia is present with hepato- and/or splenomegaly. Clinical manifestations of chronic disease result from host immune responses to schistosome eggs. *Schistosoma mansoni* and *S. japonicum* eggs most commonly lodge in the blood vessels of the liver or intestine and can cause diarrhea, constipation, and blood in the stool. Chronic inflammation can lead to bowel wall ulceration, hyperplasia, and polyposis and with heavy infections to liver fibrosis and portal hypertension (CDC: Center for Disease Control and Prevention 2018).

**Table 1:** Geographical distribution of schistosomiasis species (https://www.who.int/schistosomiasis/epidemiology/table3/en/)

Type of schistosomiasis	Species	Geographical distribution
Intestinal schistosomiasis	*Schistosoma mansoni*	Africa, the Middle East, the Caribbean, Brazil, Venezuela, Surinam
	*Schistosoma japonicum*	China, Indonesia, the Philippines
	*Schistosoma mekongi*	Several districts of Cambodia and the Lao People’s Democratic Republic
	*Schistosoma guineensis* and related *S. intercalatum*	Rain forest area of Central Africa
Urogenital schistosomiasis	*Schistosoma haematobium*	Africa, the Middle East

An estimated 85% of the world’s cases of schistosomiasis are from Africa, where prevalence rates can exceed 50% in local populations. Although schistosomiasis had been eliminated in Europe for decades, transmission of *S*. *haematobium* was reported in Corsica in 2014, when cases were identified among travelers who had swimmed in the Cavu River (Montgomery 2017).

## Case presentation

A 36-year-old Eritrean man who had lived in Denmark for the past 3 years presented to the hospital with complaints of 4 months’ history of abdominal pain and back pain, intermittent fever, productive cough with hemoptysis (just once), nausea, vomiting, and weight loss of 12 kg. The abdominal pain was mild to moderate, crampy in nature, and generalized. The patient neither had skin rash, chronic diarrhea, or rectal bleeding nor had he traveled/been to Eritrea for the past 3 years. Cardiac and pulmonary examinations were unremarkable while the abdominal exam showed signal of low-grade ascites. There was white blood cell elevation, high levels of CRP, and liver enzymes. Serologies for HIV and hepatitis virus B and C, gonorrhea, and syphilis were negative. Chest X-ray was completely normal without infiltrations or effusions. Abdominal ultrasonography reported fluid accumulation and periportal fibrosis with normal conditions in the liver and spleen and normal portal pressure. Expectorant culture and blood culture were negative for infections. Tuberculosis quantiferon PCR for differential diagnosis was positive, but direct microscopic examination of ascites fluid revealed no tuberculosis bacilli, and acid-fast stain was negative. Computed tomography (CT) of the thorax and abdomen reported normal conditions in the lungs but thickening of the terminal ileum with surrounding inflammatory change. Biopsy from the terminal ileum showed ulcer without necrotic granuloma in intestinal mucosa. Examination of stool and urine for ova was positive for Schistosoma. FDG-PET/CT scan showed enlarged lymph nodes below the diaphragm, widespread foci in peritoneum, omentum majus, and terminal ilium with increased metabolism as well as ascites with up to moderately increased metabolism.

### Clinical pathway

In our study, a diagnostic protocol including routine clinical, biochemical, microbiological, serological, and radiological examinations was performed and ultimately the patient followed up FDG-PET/CT scan in order to determine the etiology of the patient’s complex clinical feature. Patient data were retrieved from the hospital records (Table 2).

**Table 2:** The clinical pathway of the patient

Date	Tests	Results
01/05/2018	White blood cell count	Elevation
	Cardiac and pulmonary examinations	Unremarkable
	Chest X-ray	Normal
07/05	CT scan of thorax and abdomen	Thickening of terminal ileum
Early diagnosis: suspect to IBD with cholecystitis		
08/05	Ultrasonography of the abdomen	Low-grade ascites
Further diagnosis: suspect to Crohn disease		
09/05	Feces examination for TB, calprotectin, and pathogens	Negative
	Serological tests for HIV, HBV, HCV, and syphilis	Negative
	Acid-fast staining of expectorant culture and blood culture	Negative
12/05	Feces examination for intestinal and skeletal TB	Suspect
	Tuberculosis quantification PCR for differential diagnosis	Positive
Later diagnosis: suspect to intestinal TB and start of treatment		
16/05	PET/CT of whole body scan	Enlarged lymph nodes, widespread foci in peritoneum, omentum majus, and terminal ilium and ascites.
18/05	Biopsy of intestinal mucosa and ascite fluid	Ulceration, giant cell, granular
25/05	Investigation of Schistosomas ova in stool and urine	Positive only in feces
26/05	Ultrasonography of abdomen for periportal fibrosis	Positive
30/05	PCR for Schistosomas DNA of stool and urine	Positive only in feces
Final diagnosis: schistosomiasis and start of treatment		

Details are described in text

### FDG-PET/CT diagnostic procedure

**Fig. 1:**
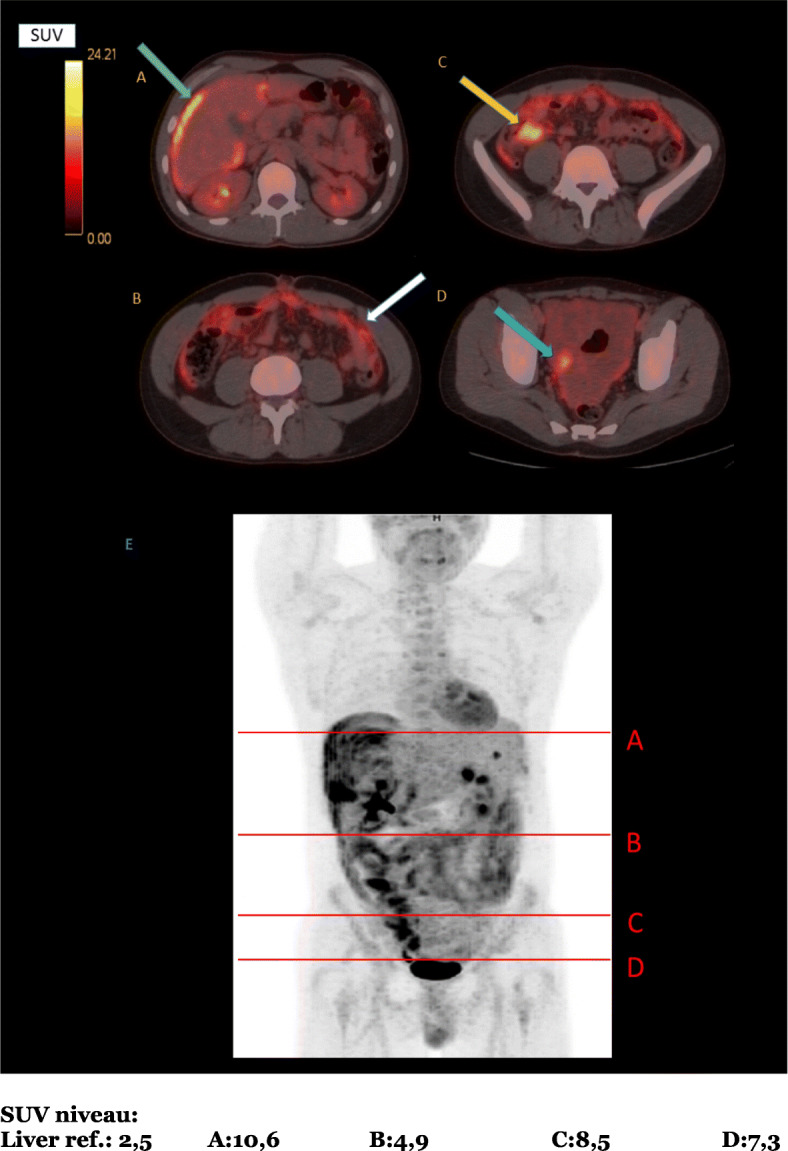
FDG-PET/CT scans showing foci with pathologic FDG-uptake. (**a**) Surface of the liver (green arrow). (**b**) Omental uptake (white arrow). (**c**) Colonic uptake (yellow arrow). (**d**) Pelvic ascites (blue arrow). (**e**) Levels of the images shown in (**a**)–(**d**) (FDG standardized uptake value scale shown)

Injected dose 18F-FDG was 224 MBq, uptake time 58 min. The PET scan was performed on a Discovery STE PET-CT scanner from GE (Chicago, IL). The PET scan was decay- and attenuation-corrected using simultaneously and reconstructed using 3D iterative reconstruction with 28 subsets, 2 iterations into 2.5-mm slices. The uptake value (SUV) was standardized using decay-corrected injected dose and total body weight and the low-dose CT (120 kV) was performed (Fig. 1).

## Discussion

Schistosomiasis is usually diagnosed by microscopic identification of parasite eggs in stool (*S. mansoni* or *S. japonicum*) or urine (*S. haematobium*). Since egg shedding may not be consistent such as in travelers and in others who have not had schistosomiasis previously, serologic tests are useful to diagnose light infections (Susan Montgomery 2017). Antibody tests do not distinguish between past and current infections. Immunologic test sensitivity and specificity vary, depending on the antigen preparation used and how the test is performed.

Positron emission tomography (PET) is a functional diagnostic imaging technique using compounds labeled with positron-emitting radioisotopes to measure cell metabolism. [18F] FDG-PET has been used to assess alterations in glucose metabolism in the brain, cancer, cardiovascular diseases, and central nervous system disorders as well as in infectious, autoimmune, and inflammatory diseases (Phelps 2000), and has been evaluated in the diagnostic make-up for fever of unknown origin (Kouijzer et al. 2018), where foci apparent on FDG-PET may not at all be visible on a contrast-enhanced CT.

E. Bueding performed the first worm physiology experiments in 1950 and found schistosomes to be demanding consumers of glucose (Bueding 1950; Salem et al. 2010). There has been only one previous report of using PET/CT to assess Schistosome burden by using in vivo imaging in mice (Salem et al. 2010). They reported that 18F-FDG-PET/CT could give clinicians and researchers a quantitative and visual tool to characterize the worm burden in infected individuals, determine the efficacy of a candidate vaccine, and provide information about parasite migration patterns and basic biology. Usually, schistosomal infections are diagnosed mainly by parasitological, serological, or molecular methods, but several imaging modalities have been employed to examine schistosomes and monitor schistosome-induced pathology in infected animals including 18F-FDG-PET/CT (Skelly 2013).

Here we are introducing a rare case of intestinal schistosomiasis in a patient with African background with 4 months’ history of nonspecific abdominal pain and 12-kg weight loss, and schistosome eggs were found in his stool and urine. Our 18F-FDG-PET/CT scan showed enlarged lymph nodes below the diaphragm, widespread foci in peritoneum, omentum majus, and terminal ilium with increased metabolism and ascites with up to moderately increased metabolism. Intestinal schistosomiasis is very scarcely reported in Europe, but it is important to take into account the differential diagnosis for acute unspecific abdominal pain and weight loss, because of increasing international traveling and disease globalization.

The patient who was diagnosed with schistosomiasis had a complicated clinical history with a severe illness. Standard clinical investigations did not reveal a definite diagnosis but raised a strong suspicion that patient had intestinal tuberculosis with unknown primary source. Eventually, in relation to the patient’s country of origin, PET/CT was used at the discretion of physician as part of clinical practice that demonstrated an enlargement of intestinal lymph nodes with increased metabolism widespread foci in peritoneum, omentum majus, and terminal ilium and ascites which was suspected for schistosomiasis infection, but a differential diagnosis could have been peritoneal carcinosis.

It should be noted that some of these tests themselves have limitations in their accuracy because there was no further sampling. In fact, we would have to be able to perform more studies in potentially more populations known to produce relevant results.

## Conclusions

We are suggesting the potential clinical contribution of FDG-PET/CT in the identification of schistosomiasis. We believe that these results are directly applicable to the typical population seen in routine clinical practice. A key question will be how they perform in different population. Studies should be designed in population with a prevalence of schistosomiasis. The classic medical discipline of detailed social and traveling history is crucial for establishing early diagnosis and treatment of exotic infective diseases, but a presumptive diagnosis of schistosome infection may be based on coincidence of FDG uptake in the intestine and lymph nodes below the diaphragm after exclusion of other causes of abdominal pain.

## Abbreviations

18F-FDG: F-18-Fluordeoxyglucose
CT: Computed tomography
PET: Position emission tomography
